# Associations between microstructural tissue changes, white matter hyperintensity severity, and cognitive impairment: an intravoxel incoherent motion imaging study

**DOI:** 10.3389/fnagi.2023.1258105

**Published:** 2023-11-28

**Authors:** Huihua Lin, Xiaomin Dai, Jiawei Su, Shengsheng Yang, Yonghong Zheng, Mingping Ma, Shun Yu

**Affiliations:** ^1^Shengli Clinical Medical College of Fujian Medical University, Fuzhou, Fujian, China; ^2^Department of Radiology, Fujian Provincial Hospital, Fuzhou, Fujian, China

**Keywords:** white matter hyperintensity, intravoxel incoherent motion imaging, diffusion weighted imaging, cognitive impairment, parenchymal diffusivity

## Abstract

**Introduction:**

White matter hyperintensities (WMHs) are a common age- and vascular risk factor-related disease and have been recognized to play an important role in cognitive impairment. However, it is still unclear what the mechanism of this effect is. In this study, intravoxel incoherent motion (IVIM) was employed to assess the microvasculature and parenchymal microstructure changes of WMHs and explore their relationship with cognitive function.

**Methods:**

Forty-nine WMH patients and thirty-one healthy controls underwent IVIM imaging, a diffusion technique that provides parenchymal diffusivity D, intravascular diffusivity D^*^, and perfusion fraction *f* . The IVIM dual exponential model parameters were obtained in specific regions of interest, including deep white matter hyperintensities (DWMHs), periventricular white matter hyperintensities (PWMHs), and normal-appearing white matter (NAWM). The independent-sample *t*-test or Mann–Whitney *U*-test was utilized to compare IVIM parameters between patients and controls. The Kruskal–Wallis test or one-way analysis of variance was used to compare IVIM parameters among DWMH, PWMH, and NAWM for patients. The Wilcoxon two-sample test or independent-sample *t*-test was used to assess the differences in IVIM parameters based on the severity of WMH. The multivariate linear regression analysis was conducted to explore the factors influencing cognitive scores.

**Results:**

WMH patients exhibited significantly higher parenchymal diffusivity D than controls in DWMH, PWMH, and NAWM (all *p* < 0.05). IVIM parameters in the three groups (DWMH, PWMH, and NAWM) were significantly different for patients (all *p* < 0.001). The severe WMH group had a significantly higher parenchymal diffusivity D (DWMH and PWMH) than mild WMH (both *p* < 0.05). The multiple linear regression analysis identified D in DWMH and PWMH as influencing cognitive function scores (all *p* < 0.05).

**Conclusion:**

IVIM has the potential to provide a quantitative marker of parenchymal diffusivity for assessing the severity of WMH and may serve as a quantitative marker of cognitive dysfunction in WMH patients.

## 1 Introduction

White matter hyperintensities (WMHs) are usually defined as bilaterally symmetrical hyperintense areas in periventricular white matter and centrum semiovale on fluid-attenuated inversion recovery (FLAIR) and T2-weighted (T2W) images (Wardlaw et al., 2013). The histopathology of WMHs mainly reflects astrogliosis, myelin, and axonal loss as a consequence of chronic ischemia caused by cerebral small vessel disease (Pantoni and Garcia, 1995; Pantoni and Simoni, 2003). Age- and vascular risk factor-related WMHs may lead to cognitive decline and dementia (Gorelick and Bowler, 2010; Pantoni, 2010; Potter et al., 2015). Hypoperfusion was found in WMHs using magnetic resonance imaging (MRI) perfusion, consistent with the above pathological findings (Markus et al., 2000). Conversely, some studies have reported that there was no significant difference in cerebral blood volume (CBV) between the WMH regions and normal-appearing white matter (NAWM) (Marstrand et al., 2002). Moreover, even increased CBV was found in some cases of WMHs (Yamada et al., 2010). Further exploration of the hemodynamic status of WMHs can help us understand the pathophysiological basis of cognitive impairment in patients with WMH.

Intravoxel incoherent motion (IVIM) MRI is a non-invasive diffusion weighted imaging (DWI) technique that can quantitatively measure microvasculature and parenchymal microstructural tissue properties (Le Bihan et al., 1988). Unlike the conventional diffusion-weighted technique in which microvascular signals confound the parenchymal signal, IVIM can separate microvascular and parenchymal MRI effects (Le Bihan et al., 1988). The purpose of the present study was to investigate the correlation between changes in microcirculation and microstructure indicated by IVIM parameters and the severity of WMH and cognitive function.

## 2 Materials and methods

### 2.1 Study participants

A prospective collection of 49 patients with WMH hospitalized in the Department of Neurology of our hospital from October 2021 to January 2023 was included. Thirty-one age- and sex-matched healthy controls were included, who were stroke-free without cognitive failures. All participants' baseline characteristics were recorded, including age, sex, and cardiovascular factors such as diabetes mellitus, hypertension, hypercholesterolemia, and smoking.

The inclusion criteria for WMH patients included WMHs seen on T2W or FLAIR, no stroke on current DWI images (except lacunar infarction), and age ≥ 60 years. The exclusion criteria included definite non-angiogenic leukoencephalopathy (such as multiple sclerosis, immune demyelination, metabolism, poisoning, infection, and tumor), suspected neurodegenerative disease other than vascular cognitive impairment (e.g., Alzheimer's disease), and a history of neurological or psychiatric disease interfering with cognitive testing, carotid stenosis of ≥50%, or a potential cardiac embolic source (e.g., atrial fibrillation). MRI imaging that showed severe head motion artifacts or with MRI contraindications was also excluded.

### 2.2 MRI acquisition

All participants underwent multimodal MRI, including T1-weighted (T1W) with voxel size = 0.7 × 0.7 × 5 mm^3^, FOV = 220 × 220 mm, TR = 1,800 ms, TE = 33 ms, slice thickness = 5 mm, T2W (voxel size = 0.7 × 0.7 × 5 mm^3^, FOV = 220 mm × 220 mm, TR = 4200 ms, TE = 94 ms, slice thickness = 5 mm), T2-FLAIR (voxel size = 0.7 × 0.7 × 5 mm^3^, FOV = 220 × 220 mm, TR = 8,000 ms, TE = 83 ms, slice thickness = 5 mm), and IVIM, on a 3.0T MRI scanner (MAGNETOM Prisma, Siemens Healthcare, Erlangen, Germany) using a 20-element head coil suitable for parallel imaging. The IVIM image was based on a diffusion-weighted spin echo single-shot echo-planar imaging pulse sequence. The specific parameters were as follows: applying gradient field in three directions perpendicular to each other, taking 12 b values (0, 50, 100, 150, 200, 250, 300, 500, 800, 1,000, 1,200, 1,500 s/mm^2^), TR = 3,600 ms, TE = 80 ms, slice thickness = 5 mm, FOV = 220 × 220 mm, and acquisition time = 4 min 23 s.

### 2.3 Image analysis

According to the research standards on small vessel diseases published by Wardlaw et al. (2013), WMHs are typically defined as bilaterally symmetrical hyperintense areas in the periventricular white matter and centrum semiovale on FLAIR and T2W. The regions of interest (ROIs) included deep white matter hyperintensities (DWMHs), periventricular white matter hyperintensities (PWMHs), and NAWM. PWMHs are defined as contiguous with the margins of each lateral ventricle and DWMHs as those separate from it (Fazekas et al., 1987; De Leeuw et al., 2000); NAWM generally is selected in the normal white matter adjacent to PWMH. The Fazekas scale rates WMHs in the periventricular and subcortical region combined on a 0–3-point scale (Fazekas et al., 1987). PWMH was graded as 0 = absence, 1 = small “caps” or pencil-thin lining, 2 = smooth “halo”, and 3 = irregular WMH extending into the deep white matter. DWMH was rated as 0 = absence, 1 = punctate foci, 2 = beginning confluence of foci, and 3 = large confluent areas. DWMH and PWMH two partial scores were added to obtain a total score (0–6 points). The severity of WMH was categorized as severe (3 + 3, 3 + 2, 3 + 1, 3 + 0, 2 + 2) or mild (0 + 1, 1 + 1, 1 + 2). Two neuroradiologists who were blinded to the clinical information of the patients used the Fazekas scale for rating WMHs. A consensus was reached through discussion in the case of inconsistent scoring results.

### 2.4 Cognitive assessment

All subjects underwent the cognitive assessment comprising of the Mini-Mental State Examination (MMSE) and the Montreal Cognitive Assessment (MoCA) (Bergeron et al., 2017) carried out by a psychiatrist who did not know the clinical and imaging data.

### 2.5 Image processing and analysis

The IVIM parametric maps were reconstructed using prototype software (Body Diffusion Toolbox 1.4; Siemens Healthineers). The IVIM signal decay was fitted with a biexponential curve using a modified two-compartment diffusion model representing a vascular and a non-vascular component (Le Bihan et al., 1988). Model fitting formula: S(*b*)/S0 = (1–f) × exp^(−*b*×*D*)^ + f × exp^[−*b*×(*D*+^^D^^*^)], where S0 represents the signal intensity at *b*-value 0 s/mm^2^, S(*b*) the signal intensity at *b*-value *b* (*b* is the diffusion factor dependent on the scanning sequence), f the microvascular perfusion fraction, D the parenchymal diffusivity, and D^*^ the intravascular (pseudo) diffusivity.

ROIs were manually segmented on IVIM images using software (imagesRadiAnt DICOM Viewer 2021) by a neuroradiologist (H.L. with 4 years of experience), who also identified and excluded infarcts and perivascular spaces (PVS). ROIs include DWMH, which is located apart from the cerebral ventricle in the subcortical white matter (uniformly selected in the center of semioval), PWMH, which is attached to the ventricular system (selected in the contiguous with the margins of each lateral ventricle), and NAWM, generally chosen in the normal white matter adjacent to PWMH. The ROI sizes varied slightly but were generally similar (area: 0.1–0.3 cm^2^), and they need to be adjusted appropriately based on the actual size of the lesion. Each ROI's mean parameters (D, D^*^, and f) were measured twice and averaged.

### 2.6 Statistical analysis

Statistical analysis was carried out using the Statistical Package for the Social Sciences (version 26.0; SPSS, IBM Corp., Armonk, NY). The distribution of quantitative variables was checked for normality and the corresponding description or method was chosen according to normality. Statistical significance was inferred for a *p*-value of < 0.05.

**Figure 1:**
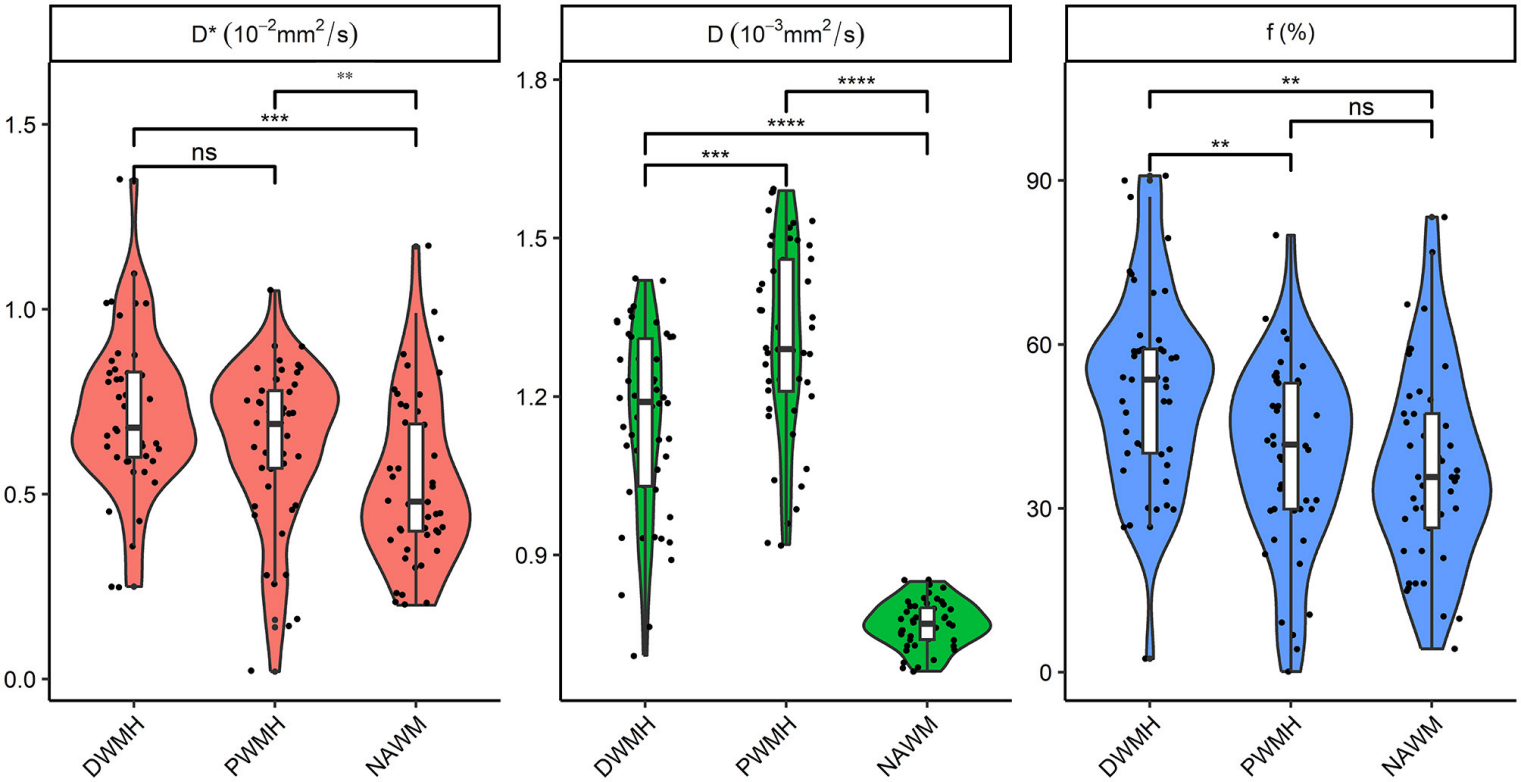
Comparison of D, D*, and f in DWMH, PWMH, and NAWM regions for patients. **P < 0.05; ***P < 0.001; ****P < 0.0001. D, parenchymal diffusivity; D*, intravascular (pseudo) diffusivity; f, microvascular perfusion fraction; DWMH, deep white matter hyperintensity; PWMH, periventricular white matter hyperintensity; NAWM, normal-appearing white matter; ns, no significance.

**Figure 2:**
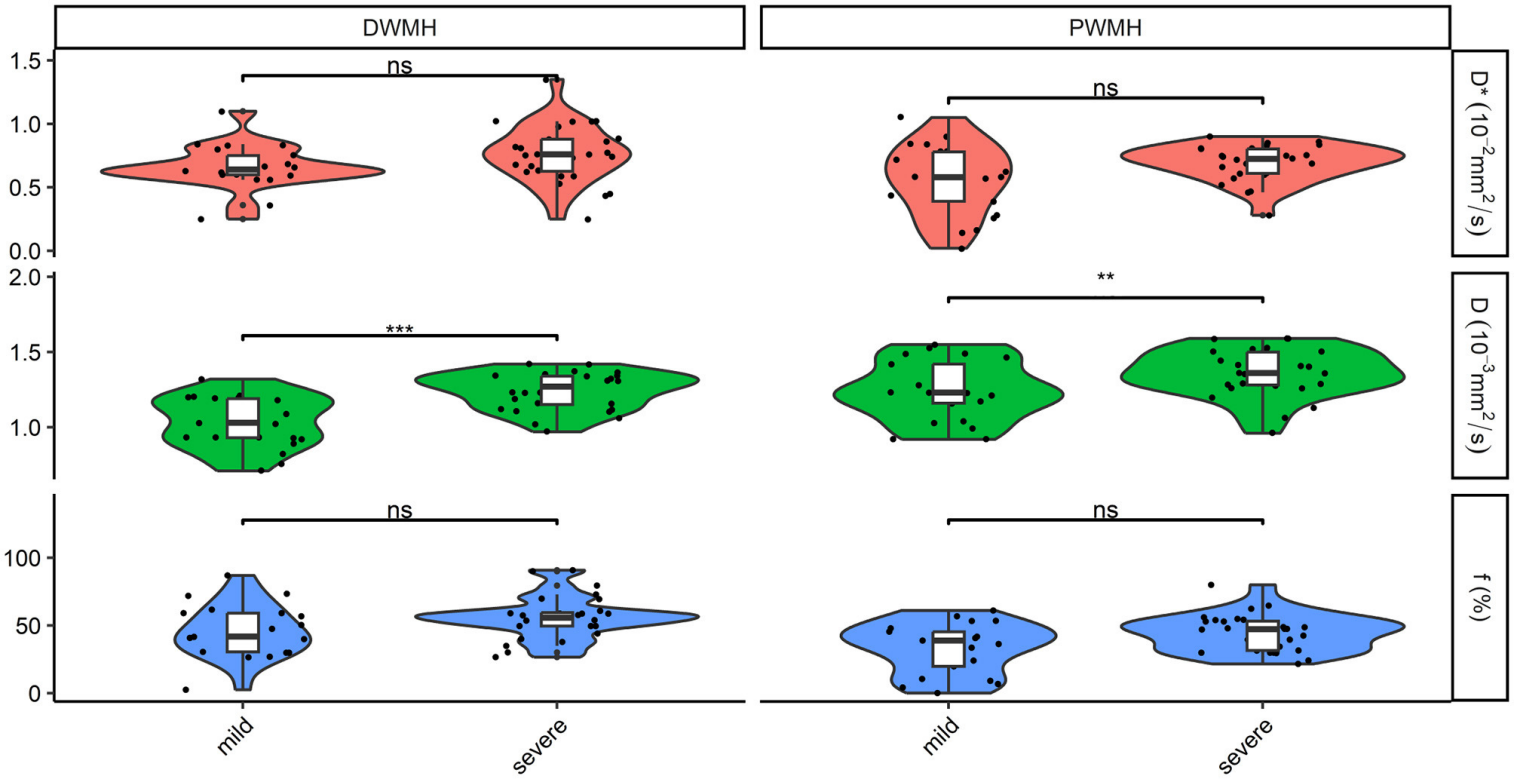
Comparison of D, D*, and f in DWMH and PWMH in patients with different severity. ***P < 0.001; **P < 0.05. D, parenchymal diffusivity; D*, intravascular (pseudo) diffusivity; f, microvascular perfusion fraction; DWMH, deep white matter hyperintensity; PWMH, periventricular white matter hyperintensity; ns, no significance.

Normally distributed continuous data were expressed as mean ± standard deviation (SD), non-parametric data as the median and interquartile range (IQR), and the counting data as *n* (%). The categorical variables were compared using the chi-square test. To compare IVIM parameters (D, D^*^, f) in different ROIs among patients and controls, we used the independent-sample *t*-test or Mann–Whitney *U*-test. Cognitive scores (MMSE, MoCA) were compared using the Mann–Whitney *U*-test among patients and controls. The Kruskal–Wallis test or one-way analysis of variance (ANOVA) test was used to compare IVIM parameters (D, D^*^, and f) among the three groups (DWMH, PWMH, and NAWM) for patients, and then, a pairwise comparison between groups was made. The significance values were adjusted by the Bonferroni correction for multiple tests. The Wilcoxon two-sample test or independent-sample *t*-test was used to compare IVIM parameters (D, D^*^, and f) between different severity of WMH. A multiple linear regression analysis was used to estimate relationships between the independent parameters and cognitive function scores (MMSE and MoCA). Variables with a *p*-value ranging between 0.1 and 0.05 were considered statistically significant in univariate analysis. The influencing factors of a *p*-value of < 0.1 on univariate analysis were further entered into the multiple linear regression model. Figures 1, 2 were made using ggplot2 software (www.rstudio.com).

## 3 Results

### 3.1 Clinical characteristics and imaging date of WMHs and controls

Table 1 summarizes the clinical characteristics of the two groups. The information about age, gender, and cardiovascular factors was described. The difference in hypertension between the two groups was statistically significant (*p* < 0.05). No significant differences in age, gender, or other cardiovascular factors (diabetes mellitus, hypercholesterolemia, and smoking) were identified among the two groups (Table 1). The results of comparison IVIM parameters (D, D^*^, and f) in various ROIs among patients and controls are also listed in Table 1. There were 2 (4.1%), 15 (30.6%), 11 (22.4%), and 21 (42.9%) patients with DWMH scores of 0, 1, 2, and 3, respectively. There were 17 (34.7%), 12 (24.5%), and 20 (40.8%) patients with PWMH scores of 1, 2, and 3, respectively. Patients had significantly higher parenchymal diffusivity D in DWMH, PWMH, and NAWM than controls (all *p* < 0.05). A lower intravascular (pseudo) diffusivity D^*^ was found in NAWM for patients compared to controls (*p* < 0.05). There were no significant differences for intravascular diffusivity D^*^ in DWMH and PWMH (*p* > 0.05). Patients had lower perfusion fraction f than controls in all ROIs, but the differences were not significant (all *p* > 0.05). Cognitive scores (MMSE and MoCA) in patients were significantly lower than in controls (both *p* < 0.05).

**Table 1:** Baseline characteristics and imaging data of WMH patients and controls.

	**WMHs (*n* = 49)**	**Controls (*n* = 31)**	***P*-value**
Age (years)	70 ± 7.7	68 ± 6.2	0.154
Male	31 (63)	19 (61)	0.859
Hypertension	40 (82)	15 (48)	0.002^**^
Diabetes mellitus	23 (47)	10 (32)	0.194
Hypercholesterolemia	14 (29)	8 (26)	0.787
Smoking	15 (31)	7 (23)	0.433
D ( × 10^−3^ mm^2^/s) in DWMH	1.19 (1.03–1.31)	0.76 (0.73–0.95)	< 0.001^***^
D^*^ ( × 10^−2^ mm^2^/s) in DWMH	0.72 ± 0.21	0.77 ± 0.16	0.239
f (%) in DWMH	51.60 ± 17.82	51.86 (44.60–60.74)	0.801
D ( × 10^−3^ mm^2^/s) in PWMH	1.31 ± 0.18	1.02 (0.86–1.28)	< 0.001^***^
D^*^ ( × 10^−2^ mm^2^/s) in PWMH	0.69 (0.55–0.79)	0.68 (0.59–0.84)	0.447
f (%) in PWMH	39.65 ± 16.63	44.95 ± 20	0.204
D ( × 10^−3^ mm^2^/s) in NAWM	0.77 ± 0.04	0.74 ± 0.03	0.003^**^
D^*^ ( × 10^−2^ mm^2^/s) in NAWM	0.48 (0.40–0.71)	0.70 ± 0.23	0.002^**^
f (%) in NAWM	37.48 ± 17.62	42.79 ± 13.39	0.155
MMSE	24 (20–27)	30 (29–30)	< 0.001^***^
MoCA	20 (16–23)	26 (25–28)	< 0.001^***^

^**^P < 0.05; ^***^P < 0.001.

DWMH, deep white matter hyperintensity; PWMH, periventricular white matter hyperintensity; NAWM, normal- appearing white matter; MMSE, Mini-Mental State Examination; MoCA, Montreal Cognitive Assessment.

Data are *n* (%), mean ± standard deviation, or median (interquartile range).

### 3.2 Comparison of IVIM parameters (D, D^*^, f) among the three groups (DWMH, PWMH, and NAWM) for patients

The Kruskal–Wallis test and ANOVA test showed that D, D^*^, and f among the three groups (DWMH, PWMH, and NAWM) had a significant difference for patients (all *p* < 0.001). Pairwise comparison showed that PWMH regions had significantly higher parenchymal diffusivity D than DWMH and NAWM (both *p* < 0.001). An intravascular diffusivity D^*^ in DWMH and PWMH was significantly higher than NAWM (both *p* < 0.05). DWMH regions had a significantly higher perfusion fraction f than PWMH and NAWM (both *p* < 0.05) (Figure 1).

### 3.3 Comparison of IVIM parameters between different severity of WMH

The severity of WMH was categorized as severe (3 + 3, 3 + 2, 3 + 1, 3 + 0, 2 + 2) or mild (0 + 1, 1 + 1, 1 + 2). Twenty-one cases (42.9%) were divided into mild WMH, and 28 patients (57.1%) were in the severe WMH group. The severe WMH group had a statistically significant higher parenchymal diffusivity D in DWMH and PWMH than mild WMH (both *p* < 0.05). There were no significant differences between different severity of WMH for perfusion fraction f and intravascular diffusivity D^*^ in DWMH and PWMH (all *p* > 0.05) (Figure 2).

### 3.4 Factors influencing cognitive scores

An MoCA score of 25 or less and MMSE score of 23–25 or less was considered a sign of cognitive impairment. A multiple linear regression analysis was used to detect which factors affect cognitive scores. A univariate analysis showed that parenchymal diffusivity D in DWMH, PWMH, and NAWM might influence the MMSE scores (all *p* < 0.1). The multiple linear regression analysis identified D in DWMH and PWMH as influencing MMSE scores (both *p* < 0.05). Regarding the MoCA score, in univariate analysis, the influencing factors included smoking, parenchymal diffusivity D (including regions of DWMH, PWMH, and NAWM), D^*^ in NAWM, and f in NAWM (all *p* < 0.1). In the multivariable linear regression analysis model, the factors influencing MoCA scores were D in DWMH and PWMH (both *p* < 0.05). Details of univariate analysis and multiple linear regression models are listed in Table 2.

**Table 2:** Univariate analysis and multiple linear regression analysis of factors influencing cognitive scores.

	**MMSE**				**MoCA**			
	**Univariate analysis**		**Multivariate analysis**		**Univariate analysis**		**Multivariate analysis**	
	β	***p*** **-value**	β	***p*** **-value**	β	***p*** **-value**	β	***p*** **-value**
Age	−0.028	0.803			0.001	0.994		
Male	−0.015	0.895			0.053	0.639		
Hypertension	−0.182	0.106			−1.442	0.153		
Diabetes mellitus	−0.068	0.550			−0.062	0.584		
Hypercholesterole-mia	−0.007	0.949			−0.050	0.662		
Smoking	−0.903	0.413			−0.199	0.076^*^	−0.081	0.400
D ( × 10^−3^ mm^2^/s) in DWMH	−0.591	< 0.001^**^	−0.424	< 0.001^**^	−0.516	< 0.001^**^	−0.282	0.022^**^
D^*^ ( × 10^−2^ mm^2^/s) in DWMH	−0.050	0.659			0.052	0.646		
f (%) in DWMH	−0.125	0.271			−0.110	0.333		
D ( × 10^−3^ mm^2^/s) in PWMH	−0.514	< 0.001^**^	−0.238	0.037^**^	−0.485	< 0.001^**^	−0.285	0.026^**^
D^*^ ( × 10^−3^ mm^2^/s) in PWMH	0.168	0.137			0.144	0.203		
f (%) in PWMH	0.076	0.501			−0.029	0.798		
D ( × 10^−3^ mm^2^/s) in NAWM	−0.312	0.005^**^	−0.081	0.403	−0.366	0.001^**^	−0.110	0.329
D^*^ ( × 10^−2^ mm^2^/s) in NAWM	0.172	0.127			0.275	0.013^**^	−0.014	0.919
*f* (%) in NAWM	0.121	0.286			0.196	0.081^*^	0.154	0.280

^*^P < 0.1; ^**^P < 0.05.

DWMH, deep white matter hyperintensity; PWMH, periventricular white matter hyperintensity; NAWM, normal-appearing white matter; MMSE, Mini-Mental State Examination; MoCA, Montreal Cognitive Assessment.

## 4 Discussion

WMHs are usually divided into two broad categories: PWMH, which is attached to the ventricular system, and DWMH, which is located apart from the cerebral ventricle in the subcortical white matter. Differences in histopathology between DWMH and PWMH are comprehensively discussed in a review by Fazekas et al. (1998). It is essential to separate two types of WMH when considering their different pathogenesis mechanisms and potential therapeutic interventions. Furthermore, NAWM, the vulnerable tissue surrounding the visible WMH, is generally chosen in the normal white matter adjacent to PWMH. Therefore, we selected DWMH, PWMH, and NAWM for ROI analysis.

In this study, we showed that WMH patients exhibited higher parenchymal diffusivity D than controls, and an increasing parenchymal diffusivity D was associated with increased risks of dementia or cognitive decline. Furthermore, parenchymal diffusivity measured with IVIM in the DWMH region showed the greatest relevancy to cognitive score. The severe WMH had higher parenchymal diffusivity D in DWMH and PWMH than mild WMH for patients.

WMH patients exhibited higher parenchymal diffusivity D than controls, which was in line with previous studies (Wong et al., 2017). It has been demonstrated that loss of tissue integrity and microstructural changes in tissue involve the loss of structural barriers, axonal injury, myelin loss, and increased extracellular space in WMH (Brown and Thore, 2011). Microstructural changes in the tissue are characterized by increased brain tissue water measured with IVIM as an increased parenchymal diffusivity D (Alexander et al., 2007). Therefore, we speculate that in the IVIM model, an increased parenchymal diffusivity D indicates irreversible microstructural damage in the corresponding brain regions. PWMH regions had higher parenchymal diffusivity compared to DWMH and NAWM for patients. Previous studies suggested increasing plasma leakage into the periventricular regions and increased blood–brain barrier permeability during aging, inducing a relatively high concentration of interstitial water in the periventricular and perivascular regions (Topakian et al., 2010; Haller et al., 2013). In contrast, the DWMH regions had a relatively low local water concentration. This may explain the higher parenchymal diffusivity D in PWMH. It was previously shown that the more severe the WMH was, the higher the ADC values of the WMH regions became (Helenius et al., 2002). We showed that the severe WMH group had higher parenchymal diffusivity D than mild WMH. This observation supports that parenchymal diffusivity increased with disease severity in WMH (Wong et al., 2017). Therefore, we suggest that parenchymal diffusivity D measured with IVIM is a promising quantitative biomarker for assessing the severity of WMH.

It was demonstrated earlier that there were hypoperfusion changes in WMH, such as decreased vascular density, tortuous arterioles, and excessive collagen deposition in veins (Alexander et al., 2007; Brown and Thore, 2011). Previous studies have compared perfusion-related parameters in IVIM with CBV and cerebral blood flow (CBF) measured by the dynamic susceptibility-contrast (DSC) MR technique (Federau et al., 2014; Wu et al., 2015, 2017). CBV and perfusion fraction f had a moderately high coefficient of correlation (Wirestam et al., 2001). Based on DSC, it was found that the perfusion in WMH was significantly reduced compared to the surrounding NAWM area (van der Veen et al., 2015; van Dalen et al., 2016). We found that patients with WMH had lower perfusion fraction f than control, indicating a decreasing microvascular volume in the corresponding area, in line with previous studies.

However, a higher perfusion fraction f was found in DWMH and PWMH than in NAWM for patients. According to the relationship between perfusion fraction f and CBV, the increasing perfusion fraction f of WMH indicates increasing microvascular volume, which may be the compensatory mechanism in the occurrence and development of WMH. An earlier study also supported this finding, suggesting that WMH was associated with a high oxygen uptake fraction and that the cerebral oxygen metabolic rate remains normal (Yamaji et al., 1997). It was previously shown that there was a significant positive correlation between CBF and perfusion fraction f in the early stages of stroke. With the establishment of collateral circulation and compensatory dilation of microvessels, perfusion fraction f was increased gradually, while CBF did not significantly change. Therefore, the increasing perfusion fraction f in WMH may be a compensatory change in microcirculation independent of CBF, and the IVIM model can contain more microvascular compensatory information than traditional perfusion imaging.

Both intravascular (pseudo) diffusivity D^*^ and perfusion fraction f are related to microvessel density and can reflect the changes in microcirculation perfusion. Our results showed that WMH patients exhibited lower intravascular diffusivity D^*^ than healthy controls. However, no significant differences existed between severe and mild WMH for D^*^. Our findings suggested that the sensitivity of intravascular diffusivity D^*^ to perfusion-related movements is lower than the perfusion fraction f, which is indicated by the IVIM model.

The clinical significance of WMH lies in its association with an increased risk of cognitive decline, motor impairment, depression, stroke, and dementia (Au et al., 2006; Prins and Scheltens, 2015). Our results showed that parenchymal diffusivity D in DWMH and PWMH was negatively associated with cognitive function assessed using MMSE and MoCA scores. An increasing parenchymal diffusivity D measured with IVIM indicates irreversible microstructural damage and loss of tissue integrity in the corresponding brain regions, associated with increased risks of dementia or cognitive decline, as shown in a previous study (van Norden et al., 2012). Previous radiologic-histopathologic studies have shown that the severity of demyelination in the postmortem tissue was positively associated with DWMH and PWMH (Haller et al., 2013). Furthermore, parenchymal diffusivity D in the DWMH region showed the greatest relevancy to cognitive score in this study. Therefore, we speculate that microstructural damage measured with IVIM in deep white matter areas (such as centrum semiovale) may play a key role in the cognitive decline of WMH patients.

Our results showed that WMH patients have an increased parenchymal diffusivity and exhibited lower intravascular diffusivity D^*^ of the NAWM than controls. However, IVIM metrics within NAWM did not appear as significant variables from the multivariate linear regression analysis. Our findings suggest that NAWM was vulnerable tissue to underlying hemodynamic and microstructural changes, which have not caused cognitive impairment for the time being. NAWM is of increasing interest as it has been shown to precede conversion into WMH over time (de Groot et al., 2013; van Leijsen et al., 2018). Before the morphological abnormalities were shown as WMH, the microstructure of NAWM was damaged. Therefore, prevention and treatment strategies targeting early changes in NAWM would be expected to be more promising and rewarding.

## 5 Limitations

First, the relationship between changes in IVIM parameters and the development of WMH was investigated only in a cross-sectional design. Second, no gold standard has been established for the assessment of WMH. This study was based on the visual rating of WMH, according to Fazekas et al. (1987), which is fast and reliable and does not require sophisticated and expensive post-processing facilities. Third, our ROl-based analysis was performed on selective WMH instead of whole volumes. Fourth, there is no unified standard for the distinctions between PWMH and DWMH; this study used the continuity rule applied in most of the visual rating scales for WMH. Fifth, based on the relatively small sample size, we defined a *p*-value of < 0.1 as “significant” in univariate analysis and further entered into the multiple linear regression model. In addition, the relatively small sample size and lack of longitudinal observation can be addressed in future studies.

## 6 Conclusion

We have shown that WMH patients had higher parenchymal diffusivity D, which was associated with increased risks of dementia or cognitive decline. Parenchymal diffusivity measured with IVIM in the DWMH region showed the greatest relevancy to cognitive score. The severe WMH group also had higher parenchymal diffusivity than mild WMH patients. IVIM has the potential to provide a quantitative marker of parenchymal diffusivity for assessing the severity of WMH and may serve as a quantitative marker of cognitive dysfunction in WMH patients.

## Data availability statement

The original contributions presented in this study are included in the article, further inquiries can be directed to the corresponding authors.

## Author contributions

HL: Writing—original draft, Writing—review & editing, Data curation, Visualization. XD: Writing—original draft, Software. JS: Writing—original draft. SYa: Investigation, Writing—original draft. YZ: Writing—original draft. MM: Project administration, Supervision, Writing—review & editing. SYu: Methodology, Supervision, Writing—review & editing.

## References

[B1] Alexander A. L. Lee J. E. Lazar M. Field A. S. (2007). Diffusion tensor imaging of the brain. Neurotherapeutics 4, 316–329. 10.1016/j.nurt.2007.05.011 17599699 PMC2041910

[B2] Au R. Massaro J. M. Wolf P. A. Young M. E. Beiser A. Seshadri S.. (2006). Association of white matter hyperintensity volume with decreased cognitive functioning: the Framingham Heart Study. Arch. Neurol. 63, 246–250. 10.1001/archneur.63.2.246 16476813

[B3] Bergeron D. Flynn K. Verret L. Poulin S. Bouchard R. W. Bocti C.. (2017). Multicenter validation of an MMSE-MoCA conversion table. J. Am. Geriatr. Soc. 65, 1067–1072. 10.1111/jgs.14779 28205215

[B4] Brown W. R. Thore C. R. (2011). Review: cerebral microvascular pathology in ageing and neurodegeneration. Neuropathol. Appl. Neurobiol. 37, 56–74. 10.1111/j.1365-2990.2010.01139.x 20946471 PMC3020267

[B5] de Groot M. Verhaaren B. F. de Boer R. Klein S. Hofman A. van der Lugt A.. (2013). Changes in normal-appearing white matter precede development of white matter lesions. Stroke 44, 1037–1042. 10.1161/STROKEAHA.112.680223 23429507

[B6] De Leeuw F. E. De Groot J. C. Bots M. L. Witteman J. C. Oudkerk M. Hofman A.. (2000). Carotid atherosclerosis and cerebral white matter lesions in a population based magnetic resonance imaging study. J. Neurol. 247, 291–296. 10.1007/s004150050586 10836622

[B7] Fazekas F. Chawluk J. B. Alavi A. Hurtig H. L. Zimmerman R. A. (1987). MR signal abnormalities at 1.5 T in Alzheimer's dementia and normal aging. Am. J. Roentgenol. 149, 351–356. 10.2214/ajr.149.2.351 3496763

[B8] Fazekas F. Schmidt R. Scheltens P. (1998). Pathophysiologic mechanisms in the development of age-related white matter changes of the brain. Dement. Geriatr. Cogn. Disord. 9(Suppl. 1), 2–5. 10.1159/000051182 9716237

[B9] Federau C. O'Brien K. Meuli R. Hagmann P. Maeder P. (2014). Measuring brain perfusion with intravoxel incoherent motion (IVIM): initial clinical experience. J. Magn. Reson. Imaging 39, 624–632. 10.1002/jmri.24195 24068649

[B10] Gorelick P. B. Bowler J. V. (2010). Advances in vascular cognitive impairment. Stroke 41, e93–e98. 10.1161/STROKEAHA.109.569921 20075354

[B11] Haller S. Kövari E. Herrmann F. R. Cuvinciuc V. Tomm A. M. Zulian G. B.. (2013). Do brain T2/FLAIR white matter hyperintensities correspond to myelin loss in normal aging? A radiologic-neuropathologic correlation study. Acta Neuropathol. Commun. 1, 14. 10.1186/2051-5960-1-14 24252608 PMC3893472

[B12] Helenius J. Soinne L. Salonen O. Kaste M. Tatlisumak T. (2002). Leukoaraiosis, ischemic stroke, and normal white matter on diffusion-weighted MRI. Stroke 33, 45–50. 10.1161/hs0102.101228 11779887

[B13] Le Bihan D. Breton E. Lallemand D. Aubin M. L. Vignaud J. Laval-Jeantet M. (1988). Separation of diffusion and perfusion in intravoxel incoherent motion MR imaging. Radiology 168, 497–505. 10.1148/radiology.168.2.3393671 3393671

[B14] Markus H. S. Lythgoe D. J. Ostegaard L. O'Sullivan M. Williams S. C. (2000). Reduced cerebral blood flow in white matter in ischaemic leukoaraiosis demonstrated using quantitative exogenous contrast based perfusion MRI. J. Neurol. Neurosurg. Psychiatr. 69, 48–53. 10.1136/jnnp.69.1.48 10864603 PMC1737001

[B15] Marstrand J. R. Garde E. Rostrup E. Ring P. Rosenbaum S. Mortensen E. L.. (2002). Cerebral perfusion and cerebrovascular reactivity are reduced in white matter hyperintensities. Stroke 33, 972–976. 10.1161/01.STR.0000012808.81667.4B 11935046

[B16] Pantoni L. (2010). Cerebral small vessel disease: from pathogenesis and clinical characteristics to therapeutic challenges. Lancet Neurol. 9, 689–701. 10.1016/S1474-4422(10)70104-6 20610345

[B17] Pantoni L. Garcia J. H. (1995). The significance of cerebral white matter abnormalities 100 years after Binswanger's report. A review. Stroke 26, 1293–1301. 10.1161/01.STR.26.7.1293 7604429

[B18] Pantoni L. Simoni M. (2003). Pathophysiology of cerebral small vessels in vascular cognitive impairment. Int. Psychogeriatr. 15(Suppl. 1), 59–65. 10.1017/S1041610203008974 16191218

[B19] Potter G. M. Doubal F. N. Jackson C. A. Chappell F. M. Sudlow C. L. Dennis M. S.. (2015). Enlarged perivascular spaces and cerebral small vessel disease. Int. J. Stroke 10, 376–381. 10.1111/ijs.12054 23692610 PMC4463944

[B20] Prins N. D. Scheltens P. (2015). White matter hyperintensities, cognitive impairment and dementia: an update. Nat. Rev. Neurol. 11, 157–165. 10.1038/nrneurol.2015.10 25686760

[B21] Topakian R. Barrick T. R. Howe F. A. Markus H. S. (2010). Blood-brain barrier permeability is increased in normal-appearing white matter in patients with lacunar stroke and leucoaraiosis. J. Neurol. Neurosurg. Psychiatr. 81, 192–197. 10.1136/jnnp.2009.172072 19710048

[B22] van Dalen J. W. Mutsaerts H. J. M. M. Nederveen A. J. Vrenken H. Steenwijk M. D. Caan M. W. A.. (2016). White matter hyperintensity volume and cerebral perfusion in older individuals with hypertension using arterial spin-labeling. Am. J. Neuroradiol. 37, 1824–1830. 10.3174/ajnr.A4828 27282862 PMC7960457

[B23] van der Veen P. H. Muller M. Vincken K. L. Hendrikse J. Mali W. P. van der Graaf Y.. (2015). Longitudinal relationship between cerebral small-vessel disease and cerebral blood flow: the second manifestations of arterial disease-magnetic resonance study. Stroke 46, 1233–1238. 10.1161/STROKEAHA.114.008030 25804924

[B24] van Leijsen E. M. C. Bergkamp M. I. van Uden I. W. M. Ghafoorian M. van der Holst H. M. Norris D. G.. (2018). Progression of white matter hyperintensities preceded by heterogeneous decline of microstructural integrity. Stroke 49, 1386–1393. 10.1161/STROKEAHA.118.020980 29724890

[B25] van Norden A. G. de Laat K. F. van Dijk E. J. van Uden I. W. van Oudheusdlen L. J. Gons R. A.. (2012). Diffusion tensor imaging and cognition in cerebral small vessel disease: the RUN DMC study. Biochim. Biophys. Acta 1822, 401–407. 10.1016/j.bbadis.2011.04.008 21549191

[B26] Wardlaw J. M. Smith E. E. Biessels G. J. Cordonnier C. Fazekas F. Frayne R.. (2013). Neuroimaging standards for research into small vessel disease and its contribution to ageing and neurodegeneration. Lancet Neurol. 12, 822–838. 10.1016/S1474-4422(13)70124-8 23867200 PMC3714437

[B27] Wirestam R. Borg M. Brockstedt S. Lindgren A. Holtas S. Stahlberg F. (2001). Perfusion-related parameters in intravoxel incoherent motion MR imaging compared with CBV and CBF measured by dynamic susceptibility-contrast MR technique. Acta Radiol. 42, 123–128. 10.1080/028418501127346459 11281143

[B28] Wong S. M. Zhang C. E. van Bussel F. C. Staals J. Jeukens C. R. Hofman P. A.. (2017). Simultaneous investigation of microvasculature and parenchyma in cerebral small vessel disease using intravoxel incoherent motion imaging. Neuroimage Clin. 14, 216–221. 10.1016/j.nicl.2017.01.017 28180080 PMC5288390

[B29] Wu W. C. Chen Y. F. Tseng H. M. Yang S.C. My P. C. (2015). Caveat of measuring perfusion indexes using intravoxel incoherent motion magnetic resonance imaging in the human brain. Eur. Radiol. 25, 2485–2492. 10.1007/s00330-015-3655-x 25693668 PMC4495260

[B30] Wu W. C. Yang S. C. Chen Y. F. Tseng H. M. My P. C. (2017). Simultaneous assessment of cerebral blood volume and diffusion heterogeneity using hybrid IVIM and DK MR imaging: initial experience with brain tumors. Eur. Radiol. 27, 306–314. 10.1007/s00330-016-4272-z 26905869 PMC5127856

[B31] Yamada K. Sakai K. Owada K. Mineura K. Nishimura T. (2010). Cerebral white matter lesions may be partially reversible in patients with carotid artery stenosis. Am. J. Neuroradiol. 31, 1350–1352. 10.3174/ajnr.A1873 20190206 PMC7965479

[B32] Yamaji S. Ishii K. Sasaki M. Imamura T. Kitagaki H. Sakamoto S.. (1997). Changes in cerebral blood flow and oxygen metabolism related to magnetic resonance imaging white matter hyperintensities in Alzheimer's disease. J. Nucl. Med. 38, 1471–1474. 9293811

